# Identifying Targets for Innovation in Amazon Reviews of Bedwetting Alarms: Thematic Analysis

**DOI:** 10.2196/43194

**Published:** 2023-07-06

**Authors:** Astha Sahoo, Savannah Leah Starr, Vadim Osadchiy, Sophia Desai, Neha Iyer, Marie Luff, Grace E Sollender, Renea Sturm

**Affiliations:** 1 David Geffen School of Medicine University of California, Los Angeles Los Angeles, CA United States; 2 Department of Urology David Geffen School of Medicine University of California, Los Angeles Los Angeles, CA United States; 3 Department of Bioengineering Samueli School of Engineering University of California, Los Angeles Los Angeles, CA United States; 4 University of California, Los Angeles Mattel Children’s Hospital Los Angeles, CA United States

**Keywords:** Amazon, bedwetting alarms, nocturnal enuresis, online reviews, early customer discovery, online, diagnosis, pediatric, teen, adolescent, sensor, treatment, age, sex, device, user, adaptability, efficacy, child health, bedwet, enuresis, Urology, pediatric urology, alarm, alert, notification, sleeping practice, sleep practice, sleep disorder, polysomnography, thematic analysis, natural language processing, NLP

## Abstract

**Background:**

Nocturnal enuresis (NE) is a frequent diagnosis in pediatric and adolescent populations with an estimated prevalence of around 15% at the age of 6 years. NE can have a substantial impact on multiple health domains. Bedwetting alarms, which typically consist of a sensor and moisture-activated alarm, are a common treatment.

**Objective:**

This study aimed to determine areas of satisfaction versus dissatisfaction reported by the parents and caregivers of children using current bedwetting alarms.

**Methods:**

Using the search term “bedwetting alarms” on the Amazon marketplace, products with >300 reviews were included. For each product, the 5 reviews ranked the “most helpful” for each star category were selected for analysis. Meaning extraction method was applied to identify major themes and subthemes. A percent skew was calculated by summing the total number of mentions of each subtheme,+1 for a positive mention, 0 for a neutral mention, and –1 for a negative mention, and dividing this total by the number of reviews in which that particular subtheme was observed. Subanalyses were performed for age and gender.

**Results:**

Of 136 products identified, 10 were evaluated based on the selection criteria. The main themes identified across products were long-term concerns, marketing, alarm systems, and device mechanics and features. The subthemes identified as future targets for innovation included alarm accuracy, volume variability, durability, user-friendliness, and adaptability to girls. In general, durability, alarm accuracy, and comfort were the most negatively skewed subthemes (with a negative skew of –23.6%, –20.0%, and –12.4% respectively), which are indicative of potential areas for improvement. Effectiveness was the only substantially positively skewed subtheme (16.8%). Alarm sound and device features were positively skewed for older children, whereas ease of use had a negative skew for younger children. Girls and their caretakers reported negative experiences with devices that featured cords, arm bands, and sensor pads.

**Conclusions:**

This analysis provides an innovation roadmap for future device design to improve patient and caregiver satisfaction and compliance with bedwetting alarms. Our results highlight the need for additional options in alarm sound features, as children of different ages have divergent preferences in this domain. Additionally, girls and their parents and caretakers provided more negative overall reviews regarding the range of current device features compared to boys, indicating a potential focus area for future development. The percent skew showed that subthemes were often more negatively skewed toward girls, with the ease of use being –10.7% skewed for boys versus –20.5% for girls, and comfort being –7.1% skewed for boys versus –29.4% for girls. Put together, this review highlights multiple device features that are targets for innovation to ensure translational efficacy regardless of age, gender, or specific family needs.

## Introduction

Nocturnal enuresis (NE) is defined as nighttime urinary incontinence in adults and children older than 5 years [1,2]. It is a common condition, affecting nearly 10% of school-age children [3]. NE can have negative consequences on pediatric mental health and academic performance [3]. Furthermore, having a child with NE can also have a negative impact on parental anxiety and depression [4].

Bedwetting alarms are the most common first-line treatment for NE. These alarms function by using moisture sensors, which provide audio or vibrating signals when detecting liquid, thereby conditioning the child to awaken when they need to urinate. Despite bedwetting alarms being the most common treatment for NE, they have several limitations. In a systematic meta-analysis comparing bedwetting alarms to medication-based therapy (desmopressin), bedwetting alarms had a higher likelihood of successfully treating NE with a lower relapse rate and better sustained response rate if patients completed the respective therapies [5]. However, nearly half of the participants treated with bedwetting alarms discontinued their use before the prescribed completion of treatment. Common reasons cited for early discontinuation included alarm discomfort, failure to awaken from sleep, the lack of efficacy, and false alarms [5]. Furthermore, an intention-to-treat analysis demonstrated that alarms did not outperform medication in achieving successful treatment [5]. This finding highlights the critical need to determine which factors limit parents and children from successfully continuing and ultimately completing treatment when using bedwetting alarms. Currently, there is insufficient data regarding product design needs that meet the real-world requirements of parents and children with NE to inform device innovation.

By analyzing consumer perspectives regarding commonly used over-the-counter health care products, top priorities for future prototypes can be identified, highlighting the importance of early customer discovery in health care innovation. “Customer discovery” is a common method used by product developers to determine whether actual customers for a product exist and what those customers desire before product development [6]; however, this method is not commonly applied to health care [6]. With the growing use of the internet to review many aspects of health care, from products to providers, this information presents a unique data source that remains underexplored. In this study, we applied early customer discovery to expand our understanding of the weaknesses and strengths of bedwetting alarms.

Using websites and social media for health care is an emerging field due to its efficiency in the advertisement and collection of data. Although internet sources such as Amazon Mechanical Turk, a crowdsourcing resource to distribute surveys and collect data [7], have been used for clinical research, there are many additional web-based sources of user-derived data that are relatively untapped in health care. For example, a recent study aimed to better understand the experiences of those struggling with male infertility by evaluating the popular discussion platform Reddit, analyzing responses on the topic using data analytics tools; the information gleaned has been applied to educate health care practitioners on the current concerns of the male infertility community [8]. Specific to consumer-marketed health technology, web-based reviews and social media platforms foster competition that has diversified the range of products and product features available by catering to different user demographics beyond those commonly participating in clinical research trials [9].

This study arose from a recent partnership between a medical student innovation program and a capstone engineering course at a research university. First, a stepwise biomedical innovation needs–mapping process was performed as part of a formal medical innovations program, Sling Health [10]. During this program, perspectives were obtained from various stakeholders, including patients and their families as well as health care staff through clinical observation [10]. The need for more efficient treatments for NE was identified as the top unmet need in pediatric urology [10]. Based on this finding, a team with backgrounds in bioengineering and medicine was assembled to create a prototype to meet this need. Further analysis of the strengths and deficits in current products was required to expand our understanding of needs across a range of settings, parent-child dynamics, and characteristics.

The primary aim of this study was to elucidate areas of satisfaction and dissatisfaction with the current alarm-based treatment of NE through a systematic evaluation of consumer reviews of bedwetting products, with a focus on defining specific targets for future innovation. Secondary analyses by gender and age were also performed to determine whether reported experiences differed between specific user groups.

## Methods

### Data Extraction

**Figure 1:**
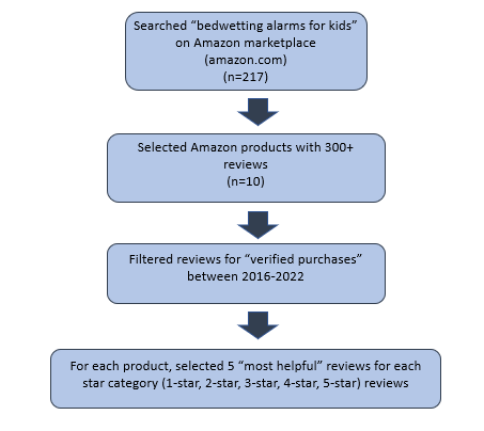
Method for extracting Amazon reviews of bedwetting alarms.

Using the search term “bedwetting alarms for kids,” Amazon reviews from 2016 to 2022 were extracted from the Amazon marketplace [11] for all bedwetting products that had over 300 ratings in February and March 2022. Data was collected manually by 4 evaluators. Reviews were extracted if they met three criteria: (1) the review was posted within the target date range; (2) the review length was >5 words; and (3) Amazon verified that the reviewer had purchased the product. Amazon user product ratings occur on a 1- to 5-star scale; thus, reviews are categorized into 5 “star categories”: 1-star, 2-star, 3-star, 4-star, or 5-star reviews. To further target a range of experiences, up to 5 reviews from each star category, for a maximum of 25 reviews per product, were analyzed. In the Amazon marketplace, users can mark a post as “helpful” if the review helped them decide whether to purchase the product or if the review helped them fix a problem they were having with the product [12]. To collect data that were in accordance with most users, the 5 reviews with the greatest number of “helpful” marks were collected for each star category (Figure 1). For each review that met these criteria, the following data were analyzed further: the date of publication, review title, and review content.

### Ethical Considerations

All data were publicly available and did not involve human subjects. Therefore, this study was exempt from review by the Institutional Review Board of the University of California, Los Angeles.

### Qualitative Thematic Analysis

A free-text and qualitative thematic analysis was completed through manual annotation of selected reviews. Meaning extraction method, a technique that forms simple overarching themes from text by extracting content words (ie, nouns, verbs, and adjectives) and removing connective words (ie, articles, prepositions, and pronouns), was applied to identify major themes and subthemes for each review [13]. Themes that related to unique categories of strength and deficits faced by users of bedwetting alarms were identified, and subthemes defined these further. A minimum of 2 investigators, including the research mentor, reviewed posts during data collection to identify a preliminary set of common themes. These themes were then discussed in depth among all 4 investigators to achieve consensus. The team of investigators then formed subthemes during the process of evaluation. After an iterative process evaluating all reviews, themes and subthemes were discussed and finalized. For each analyzed review, investigators rated the discussion of each subtheme on a 3-point scale, where –1 indicated that the reviewer had a negative experience regarding that subtheme, 0 indicated that the experience was neutral or not discussed, and 1 indicated that the reviewer had a positive experience with regard to that subtheme. After discussion, a minimum of 2 reviewers agreed upon each rating. All subthemes were ranked from most negative (weakness) to most positive (strength) in current NE products using the percent skew, which was calculated by taking the sum of all investigator ratings and dividing by the total number of reviews for each product.

In addition to major themes, relevant information, such as price and demographics of the child for whom the device was purchased, and the follow-up review were collected. Specifically, the gender and age of the child using the device were included in data collection and analysis if provided in the review. Subanalyses by gender (divided into boy and girl cohorts) and age (divided into 2 cohorts aged 3-8 and 9-12 years) for those reviews that provided this information were completed using percent skew. Of note, there were no ages mentioned outside the age ranges in these cohorts.

### Consideration of Researcher Characteristics, Reflexivity, and Mitigation of Biases

Four investigators, with at least 2 investigators evaluating each review, were involved in the Amazon review evaluation process. One reviewer was a third-year medical student, one reviewer was a second-year neuroscience undergraduate, and 2 reviewers were bioengineering undergraduate students. The inherent potential for bias between bioengineering students was recognized. To prevent this bias and provide varied perspectives in the analysis of each review, the bioengineering students were paired with either the medical or neuroscience student whose clinical experience allowed the completion of the initial thematic mapping. When discrepancies arose, consensus was reached through a wider complete team review with the research mentor, who is a pediatric urologist.

## Results

Of the 136 bedwetting alarm products identified in total, 10 were selected for further analysis based on the provided selection criteria. Products had an average of 1693 (range 329-4380) posted reviews with a mean overall rating of 3.9 (range 3.2-4.5) out of 5. The median price of products was US $68.5 (range US $33.99-US $299). A total of 250 reviews (25 for each product as described previously) were analyzed.

The 4 most frequently identified themes were long-term concerns, marketing, alarm characteristics, and device mechanics and features. Within these overarching themes, distinct subthemes emerged. For long-term concerns, reviewers discussed overall effectiveness, proper use, and durability. In terms of marketing, parents remarked on the cost-to-value ratio and customer service support available for the product. Alarm features and accuracy were common subthemes described regarding alarm characteristics. For device mechanics and features (excluding the alarm or sound), parents commented on specific device features, comfort, safety, ease of use, and effectiveness of reinforcement tools such as reward charts that accompanied certain bedwetting alarms (Table 1).

The total number of mentions for each subtheme was evaluated. The most-mentioned theme was long-term concerns (468 total mentions), and the most-mentioned subtheme was effectiveness (164 [mentions]/250 [total reviews], 65.6%). Marketing was the least-mentioned theme (78 total mentions), and proper use was the least-mentioned subtheme (6/250, 2.4%; Table 2). Representative quotes for each theme and subtheme are illustrated in Table 2. Although most subthemes had positive and negative mentions, there were 2 subthemes, safety and durability, that only had negative mentions (6/250, 2.4% and 67/250, 26.8%, respectively). Therefore, for safety and durability, no positive representative quotes were available (Table 2).

**Figure 2:**
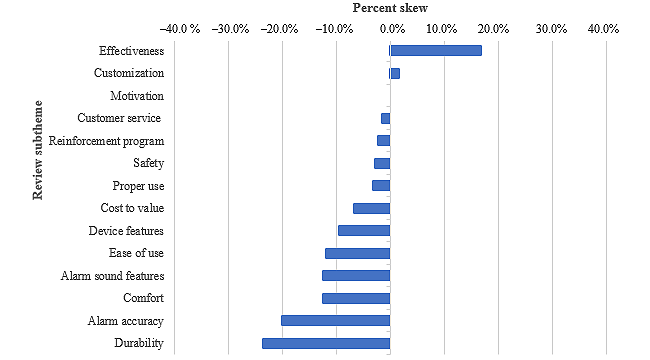
Percent skew of subthemes identified in Amazon reviews of bedwetting alarms.

Figure 2 demonstrates the overall skew of each identified subtheme. The percent skew was calculated by summing the total number of mentions of each subtheme, +1 for a positive mention and –1 for a negative mention, and dividing this sum by the total number of reviews. Device subthemes that were positively skewed included effectiveness (42 [total composite score]/250 [total reviews], 16.8%) and the ability to customize a device (4/250, 1.6%). The most negatively skewed subtheme was the durability of the alarm (–59/250, –23.6%), followed by alarm accuracy (–50/250, –20%), comfort (–31/250, –12.4%), and alarm sound features (–31/250, –12.4%), thereby indicating that reviewers more commonly had negative experiences with these features.

**Table 1:** Identified themes and subthemes through thematic analysis of Amazon reviews of bedwetting alarms.

Theme	Subtheme
Long-term concerns	Proper use Durability Effectiveness
Marketing	Cost to value Customer service
Alarm characteristics	Alarm accuracy Alarm sound features
Device mechanics and features	Device features Safety Comfort Reinforcement Ease of use

**Table 2:** All identified themes and subthemes with the number of mentions and representative quotes.

Theme and subtheme			Mention (N=250 reviews), n (%)		Positive quote		Negative quote
**Long-term concerns**							
	Effectiveness	164 (65.6)		“We are 10 weeks into the program and he has not had any accidents for 7 weeks! We also sit down and watch the program and check in videos together. The star system was also very motivating for our son. I would give this program 5 stars for effectiveness. It is a process but again, for our family, totally worth it for the quick results!”		“We have tried this alarm for 3 years now and it does nothing for our son. I am exhausted at trying to find things to stop his night time enuresis. I thought this would be the game changer, but the only thing it did was wake us up”	
	Durability	67 (26.8)		No positive quotes available		“Worked for about a month and all alarms stopped working. Ordered another probe because probes are first to die. Changed batteries also. No alarm (vibrate/audible) with new probe and new batteries”	
	Proper use	6 (2.4)		“For any review that says their child won’t wake up with the alarm and they won’t get out of bed, you have to sleep in their room to make sure they get up. That’s half the battle. If the alarm isn’t working, it’s most likely user error. If you do it right, we are proof that it’ll work”		“The first night, I realized the cord wasn't in the alarm properly (it requires an extra step where you turn it 90 degrees). The 2nd night, we used vibration only and my son doesn't think the alarm went off even though he wet (or maybe it did then stopped without waking him? no idea). The 3rd night, we tried both music and vibration, and again it either didn't go off or didn't wake him when he wet. Trying again tonight with it on full volume.”	
	Effectiveness	164 (65.6)		“This literally was worth every penny”		“We paid several hundred dollars for this product and all we got for our money was months of sleepless nights and a shorted out non working pad”	
	Durability	67 (26.8)		“If you want to help your child with nighttime dryness, purchase this product and you’ll get a team who also cares about your child to help you.”		“I tried calling the number for the company several times but only got voicemail, and messages left on it weren't replied to. I'm buying a similar device from a different company.”	
**Marketing**							
	Cost to value	64 (25.6)		“I like the fact that it has multiple sounds. That way if your child has become familiar with a sound you can change it. The volume is great and I can hear it in my room across the hall.”		“Shocked at how loud this alarm is, with no volume control. It woke up the entire house and my 9-year-old was hysterical!”	
	Customer service	14 (5.6)		“We attached it to the front of his underwear and just the slightest drop of liquid sets it off. It's loud and won't quit till he unclips it which is good.”		“Tried it and it does not go off. On occasion, it would go off, but way too late.”	
**Alarm characteristics**							
	Alarm sound features	88 (35.2)		“He liked this one since it was a mat under the sheet and not something attached to his clothing.”		“My 8 year old wore this for two nights, but refused to wear it anymore. Says it’s not comfortable and itchy.”	
	Alarm accuracy	79 (31.6)		“We really like that the sensor and alarm are separate pieces and that the piece that wakes you up is not an arm band.”		“We had this product for 30 days before it broke. The wiring pulled out from the alarm part.”	
**Device mechanics and features**							
	Comfort	39 (15.6)		No positive quotes available		“In the middle of the night my son screamed loudly, we immediately ran to his room and disconnected the alarm. He told us that the alarm was giving him shocks and noticed that his entire area was red. We applied ice packs on him with little to no results we had to visit our doctor in the morning.”	
	Device features	30 (12)		“Pros...It comes with stickers and log book.”		“…[T]he reward stickers are insensitive and humiliating. Stickers of a baby in diapers!!! How insulting! And if they pee the bed, they’re supposed to use a sticker of a crying baby in a diaper.”	
	Safety	9 (3.6)		No positive quotes available		“Worked for about a month and all alarms stopped working. Ordered another probe because probes are first to die. Changed batteries also. No alarm (vibrate/audible) with new probe and new batteries”	
	Reinforcement	7 (2.8)		“For any review that says their child won’t wake up with the alarm and they won’t get out of bed, you have to sleep in their room to make sure they get up. That’s half the battle. If the alarm isn’t working, it’s most likely user error. If you do it right, we are proof that it’ll work”		“The first night, I realized the cord wasn't in the alarm properly (it requires an extra step where you turn it 90 degrees). The 2nd night, we used vibration only and my son doesn't think the alarm went off even though he wet (or maybe it did then stopped without waking him? no idea). The 3rd night, we tried both music and vibration, and again it either didn't go off or didn't wake him when he wet. Trying again tonight with it on full volume.”	

**Figure 3:**
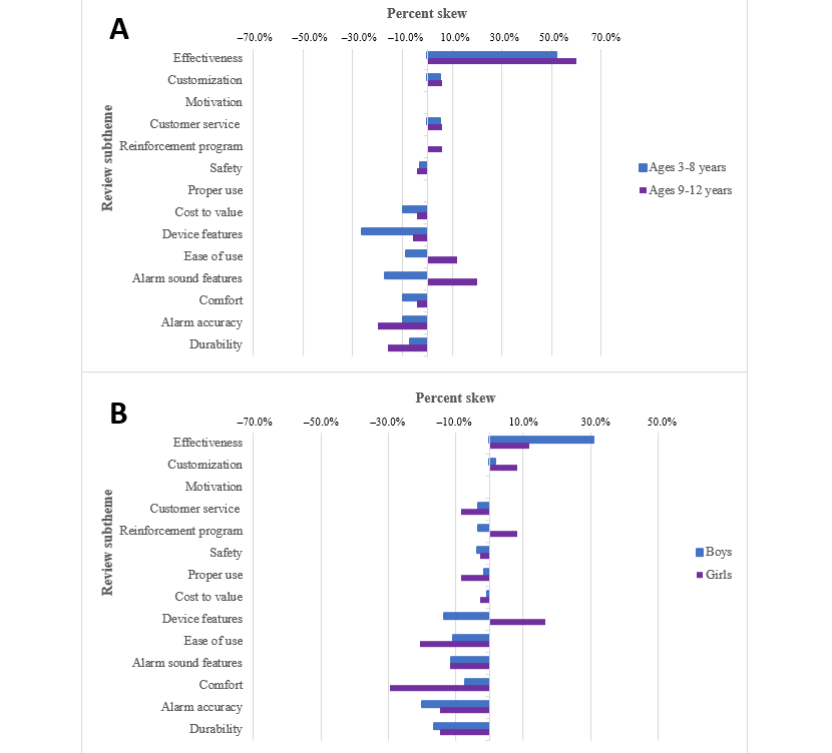
Percent skew subanalysis by age and gender: (A) ages 3-8 years versus 9-12 years and (B) boys versus girls.

Additional data analysis revealed 2 specific subpopulations with more negative experiences compared with the overall reviews of current products: younger age and identification as a girl. Of the 250 total reviews, 95 mentioned the age of the child (70 with children aged 3-8 years and 25 with children aged 9-12 years). After analyzing for age, the subtheme alarm sound features had a positive skew for older children (5/25, 20%) compared to a negative skew for younger children (–12/70, –16.9%). Additionally, the subtheme ease of use had a negative skew for younger children (–670, –8.5%) compared with a positive skew for older children (5/25, 20%). Finally, the subtheme device features more negatively affected younger children (–18/70, –26.3%) than older children (–1/25, –4%; Figure 3A). A total of 175 reviews mentioned the gender of the child (141 boys and 34 girls). In the gender subanalysis, specific subthemes emerged as more negatively skewed for girls compared with boys. Specifically, effectiveness (43/141, 30.7% for boys and 4/34, 11.8% for girls), ease of use (15/141, –10.7% for boys and –7/34, –20.5% for girls), and comfort (–10/141, –7.1% for boys and –10/34, –29.4% for girls) demonstrated differences between boys and girls (Figure 3B).

The evaluation of consumer reviews also revealed different opinions stratified by gender regarding specific device features, including alarms with long cords, wireless alarms with a sensor in the underwear, alarms with arm bands, and sensor pads. In the evaluation of these specific device features by gender, alarms with long cords (–1/4, –25% for girls and –1/10, –10.0% for boys), arm bands (–1/3, –33% for girls and 1/30, 3.3% for boys), and sensor pads (–1/12, –8.3% for girls and 0/15, 0% for boys) negatively impacted girls more than boys. Perceptions of wireless alarms had no skew for either gender (0/17, 0% for girls and 1/63, 1.6% for boys; Multimedia Appendix 1).

## Discussion

### Principal Findings

To improve satisfaction and compliance with bedwetting alarms, our study evaluated consumer perceptions of currently available products. The evaluation of bedwetting alarm reviews on Amazon provided the identification of specific targets that need to be addressed. Our study was developed following a recent qualitative study performed by our group. Through 6 weeks of clinical immersion, which involved both clinical observation and interview-based insight extraction across all members of the treatment and patient care team in the pediatric urology clinic, we identified challenges with current bedwetting alarms as the top unmet need [10]. Consistent with our single-center needs mapping, the cause of bedwetting alarm inefficacy and dissatisfaction across a range of products was dissatisfaction with and challenges presented by certain device features as identified in Amazon reviews. From this evaluation of consumer experiences, we have identified 3 prominent targets for innovation: the availability of a wider range of alarm sounds, including more volume and tone options; improved user-friendliness of design; and the incorporation of preferred features across genders. Findings from this study add granularity to the challenges experienced across current solutions. In doing so, our findings provide innovators a roadmap for developing subsequent prototypes for clinical testing. Likewise, our findings will inform clinicians of potential challenges to address with families when prescribing bedwetting alarms to maximize device acceptance and use and will provide children and their families an overview of key features that may impact effective use.

Experience with alarm sound features was negatively skewed in our study, suggesting that more diverse sound options are needed for alarms to accommodate different children and family needs while minimizing alarm fatigue. In many reviews for one product in particular, there was a mixture of complaints regarding volume. For example, one reviewer stated “[the] alarm does not even remotely stir my son,” whereas another reviewer for the same product complained that the alarm was “obnoxiously and blaringly loud.” Reviewers frequently expressed a desire for more options, including different volume options, vibration options, and various song and nonsong alarm tones. Although we found that parents are seeking more sound options, innovators in this space should also account for the evidence that particular sounds may be more effective at awakening children [14]. A recent study that evaluated sound options for residential fire alarms found that low-frequency tones, the voice of a female stranger, and the voice of the child’s mother were significantly more effective at waking children than high-frequency tones [14]. This is consistent with the findings of a systemic review on the effectiveness of different alarm types on sleep inertia [15]. Based on our findings and prior evidence, bedwetting alarms require more alarm tone and volume options, and it is recommended that device developers ensure that low-frequency and voice tones are incorporated to maximize their effectiveness.

Negative experiences with comfort and the ease of use were also identified, indicating a need for improved user-friendliness for bedwetting alarms. This has been previously described as being essential for successful treatment [5,16]. Although certain complex device features may have been added primarily to drive sales volume, such features may in fact impede routine use and may increase the likelihood of early discontinuation, thereby limiting clinical effectiveness [16]. Specific device features that were discussed in reviews included the negative impact of devices with multiple versus fewer components. In particular, products that required multiple steps to turn off and set the alarm received less favorable reviews. Based on these findings, it is recommended that the development of easy-to-use, essential options be a primary focus while keeping the number of separate pieces of equipment to a minimum.

Although more diverse sound options for alarms and improved user-friendliness were themes that have been previously discussed in the literature [5,16], we describe the importance of the incorporation of girl-friendly features, which was frequently noted as a unique target area. Across all bedwetting devices analyzed, girls specifically had more negative skew in many of the major themes identified than boys, particularly with the ease of use and comfort. One reviewer stated, “directions are geared toward boys, so we had to improvise the placement of the sensor,” and another reviewer for a different product stated, “[the product] really needs a redesign for usability by young children, especially girls.” In multiple reviews, parents stated that the placement of the sensor was especially geared toward boys, and “large, stiff” cords and alarms between the legs of young girls made the use of the product uncomfortable. From our study, we demonstrated that girls were more negatively impacted by device features such as long cords, arm bands, and sensor pads. A potential indirect cause of this variant experience between genders may be the lack of equal gender representation in prior clinical studies of bedwetting alarms. In a recent systemic review on alarm use for the treatment of NE, of the 5026 participants in the 74 studies evaluated, only 33% of participants were girls [16]. Moreover, little is known about gender variance in prototype testing. The lack of female representation in these studies and their inclusion of predominantly male patients could be a potential contributor to the perceived lack of preferred features by girls currently present in bedwetting alarms. These findings highlight the importance of including equal gender representation in future prototype testing and clinical studies. By ensuring the inclusion of girls in future studies and prototype testing from our engineering team, girl-friendly features will be readily incorporated into developing bedwetting alarms.

Through the process of early customer discovery used in this study, we have identified key targets for improvement in future bedwetting alarm prototypes. Analyzing Amazon reviews enabled the research team to assemble a wide range of patient opinions to drive innovation. In the past, Amazon reviews have also been applied in health care to understand marketing myths [17] and patient experiences of uncommonly used treatment modalities [18] and to evaluate product efficacy and safety [19]. However, to our knowledge, this is the first such study for bedwetting alarms. This underutilized data source can enrich our understanding of health care by improving our understanding of the lived patient experience associated with the home use of commercially available health care products.

### Limitations

Limitations of this study include the use of a limited data set, potential for implicit bias, and product selection. First, we were limited by solely analyzing the reviews of customers who voluntarily wrote a review. These reviews may have been skewed by those who had a more negative experience, since individuals are more likely to highlight and express their negative views on an experience rather than their positive views [20]. Furthermore, implicit bias could have affected the reviewer team, who were aware of the star rating of the product when extracting language for analysis. However, regardless of the rating, reviewers often had negative and positive perceptions about different aspects of the product. By evaluating each product feature independently, bias and subjectivity from the star rating were mitigated. Finally, analyses were limited to the device and patient features that authors chose to include in each review, and a limited number of products were analyzed. Out of 136 products, 10 common products were reviewed, representing 7% of bedwetting alarm products on the Amazon marketplace. The selection criteria for these devices could have contributed to selection bias for those most commonly purchased. Furthermore, our data set was limited by only selecting bedwetting alarm products on Amazon rather than alternative marketplaces. Put together, this study represents products with a range of common features. Future studies are needed to include a wider range of reviews and devices, potentially aided by automated analysis such as natural language processing and targeted feature analysis to further inform future device development.

### Conclusions

This study analyzed Amazon reviews, both validating and expanding upon our qualitative findings in the clinical setting regarding an unmet need for feature innovation in bedwetting alarm systems. To improve satisfaction and compliance with bedwetting alarms, our results provide an innovator roadmap for subsequent device innovation. Our results suggest that more variance in alarm sound features would be beneficial to children and families to improve device compliance, as children of different ages have different preferences in terms of volume and the type of alarm. Less complexity in devices is suggested, as added device features such as arm bands and lengthy cords resulted in devices that limited consistent, effective, and prolonged use. Additionally, current device features may impact girls more negatively overall than boys, indicating that devices were not specifically optimized across genders. Increased representation of girls in device testing is needed. Subthemes such as durability and comfort further suggest that the optimal device should focus on child-friendly features, allowing for wear and tear while ensuring patient comfort. Future prototype evaluation should prioritize these identified device features to optimize translatability and clinical effectiveness. In addition to pinpointing areas necessary for optimization and improvement, this study has highlighted the value of marketplace reviews and the information they can provide to researchers analyzing clinical products developed for home use.

## Appendix

Multimedia Appendix 1 Percent skew analysis by gender for specific device features of bedwetting alarms identified in Amazon reviews.

## Abbreviations

NE: nocturnal enuresis

## Data Availability

The data sets generated during and/or analyzed during the current study are available from the corresponding author on reasonable request.
