# Supplementary material for: Identifying Targets for Innovation in Amazon Reviews of Bedwetting Alarms: Thematic Analysis
Source: Interact J Med Res. 2023 Jul 6;12:e43194. doi: 10.2196/43194 (PMC10360013; doi:10.2196/43194)
Supplement: Multimedia Appendix 1 [file ijmr_v12i1e43194_app1.docx]

Figure S1: Percent skew analysis by sex of specific device features of bedwetting alarms identified in Amazon reviews. A. Vibrating sensor on underwear with a cord connecting to an alarm on the shirt. B. Wireless alarm with sensor on underwear. C. Sensor Pad. D. Alarm on an arm band with a sensor on the underwear.

–40.0% –30.0% –20.0% –10.0% 0.0% 10.0% 20.0% 30.0% 40.0%

–40.0% –30.0% –20.0% –10.0% 0.0% 10.0% 20.0% 30.0% 40.0%

–40.0% –30.0% –20.0% –10.0% 0.0% 10.0% 20.0% 30.0% 40.0%

–40.0% –30.0% –20.0% –10.0% 0.0% 10.0% 20.0% 30.0% 40.0%
